# Pilot implementation of a sonography simulator in gynecological medical training and continuing education: A practical report

**DOI:** 10.3205/zma001713

**Published:** 2024-11-15

**Authors:** Anne Röhle, Marie-Christin Willemer, Cahir Birdir, Katharina Nitzsche

**Affiliations:** 1 TUD Dresden University of Technology, Carl Gustav Carus Faculty of Medicine, Institute of Medical Education, Medical Interprofessional Training Centre (MITZ), Dresden, Germany; 2 University Hospital Carl Gustav Carus at the Dresden University of Technology, Clinic and Out-patient Department for Obstetrics and Gynecology, Dresden, Germany

**Keywords:** sonography, simulator, feedback, e-Learning, medical education, medical training, skills lab

## Abstract

**Objective:**

In addition to patient consent, learning sonography requires considerable time and personnel resources. To implement patient-friendly and resource-saving ultrasound teaching, a comprehensively equipped sonography simulator (SoSim) was purchased at the Medical Interprofessional Training Centre (MITZ) of the Faculty of Medicine at TU Dresden. In a first step, the SoSim training was trialed in a sample (n=5) in cooperation with the Clinic and Polyclinic for Gynecology and Obstetrics at Dresden University Hospital (GYN). Based on the findings, the aim is to extend the project to University Medicine Dresden.

**Description of the project:**

After creating the necessary structural and organizational conditions, five female GYN trainees trained two defined modules in SoSim on transvaginal and abdominal ultrasound. The evaluation was carried out by online-based evaluation of the participants and process analysis.

**Results:**

The process analysis showed that close supervision of the learners is required and that booking individual appointments is time-consuming. The evaluation of the participants showed a positive mean change in the approval ratings for 11 out of 14 competency-based questions.

**Conclusion:**

Despite the integrated SoSim learning program, support for learners is necessary. By piloting the use of SoSim to teach transvaginal ultrasound as part of gynecological specialist training, University Medicine Dresden is a pioneer in innovative and patient-friendly teaching. The pilot project has laid the foundations for the expansion of the project into other areas.

## Zusammenfassung

**Zielsetzung:**

Das Erlernen der Sonografie erfordert neben dem Einverständnis der Patient*innen erhebliche zeitliche und personelle Kapazitäten. Um patientenentlastende und ressourcenschonende Ultraschalllehre zu realisieren, wurde im Medizinischen Interprofessionellen Trainingszentrum (MITZ) der medizinischen Fakultät der TU Dresden ein umfassend ausgestatteter Sonografiesimulator (SoSim) angeschafft. In einem ersten Schritt wurde in Kooperation mit der Klinik und Poliklinik für Frauenheilkunde und Geburtshilfe des Universitätsklinikums Dresden (GYN) in einer Stichprobe (n=5) das SoSim-Training erprobt. Basierend auf den Ergebnissen wird die Ausweitung des Projekts auf die Hochschulmedizin Dresden angestrebt.

**Projektbeschreibung:**

Nach Schaffung der notwendigen strukturell und organisatorische Bedingungen trainierten fünf Berufseinsteigerinnen der GYN zwei definierte Module im SoSim zum transvaginalen und abdominalen Ultraschall. Die Auswertung erfolgte durch onlinebasierte Evaluation der Teilnehmenden und Prozessanalyse.

**Ergebnisse:**

Die Prozessanalyse zeigte, dass es einer engen Begleitung der Lernenden bedarf und eine individuelle Terminbuchung zeitintensiv ist. In der Evaluation der Teilnehmenden zeigte sich bei 11 von 14 kompetenzorientierten Fragen eine positive Mittelwertveränderung der Zustimmungswerte.

**Schlussfolgerung:**

Trotz integriertem SoSim-Lernprogramm ist eine Begleitung der Lernenden notwendig. Die Hochschulmedizin Dresden ist mit der Pilotierung des Einsatzes des SoSim zum Erlernen des transvaginalen Schalls im Rahmen der gynäkologischen Facharztausbildung Wegbereiter für innovative und patientenschonende Lehre. Mit der Pilotierung wurden Grundlagen für die Ausweitung des Projekts in andere Bereiche geschaffen.

## 1. Background

Sonography is firmly integrated into preventive, routine, emergency, and follow-up diagnostics. Standardized and structured training of all medical professionals is therefore essential, while at the same time conserving human, spatial and financial resources [1].

At the Medical Interprofessional Training Centre (MITZ) of the Faculty of Medicine at TU Dresden (MFD), students of medicine, dentistry and midwifery train basic practical skills and communication skills. The MITZ also offers opportunities for further and advanced training.

Since 2015, ultrasound teaching at the MITZ has been successively implemented, expanded and professionalized. During the collaboration with the Clinic and Polyclinic for Gynecology and Obstetrics at Dresden University Hospital (GYN) in the midwifery degree program, points of intersection became clear regarding efforts to ensure resource-conserving, needs-oriented teaching and the bundling of didactic skills. Limitations in current sonography training due to limited space and personnel capacities became apparent in both areas, as well as the need for patient-friendly teaching.

**Figure 1:**
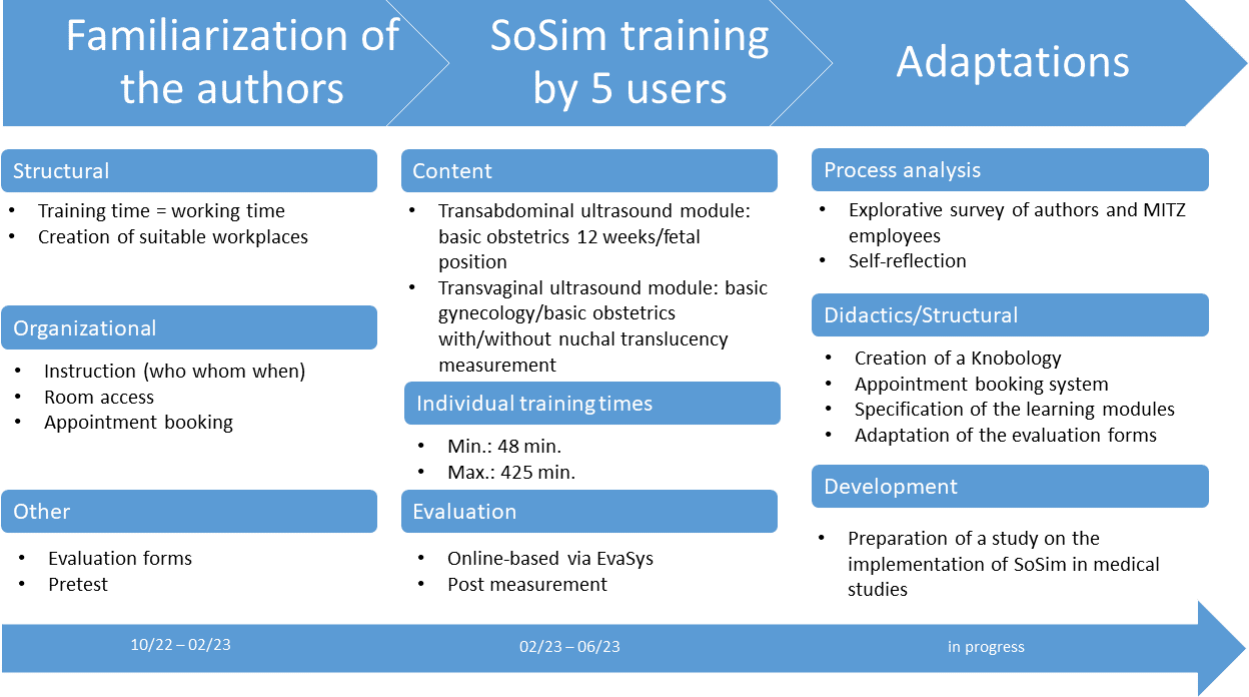
Time and content aspects of the project with necessary structural and organizational conditions and adjustments for practical implementation

In 2022, the MITZ purchased a fully equipped sonography simulator (SoSim) with the support of the MFD: the SCANTRAINER 7A of Skills Med Germany. In the pilot phase, the SoSim was tested in cooperation with the GYN. Under the technical and didactic supervision of the authors, five young professionals from GYN trained two defined modules on the SoSim over a period of five months and were asked about their subjective appraisal after the training (see figure 1 (Fig. 1)). The aim was to use the pilot phase to identify positive effects in terms of competence gain and to create a spatial and organizational basis for a sustainable and comprehensive use of the SoSim for entire University Medicine Dresden.

## 2. Description of the project

Initially the authors were familiarized with the SoSim, and the necessary structural and organizational conditions were created to adequately support the users learning progress (see figure 1 (Fig. 1)). 

**Figure 2:**
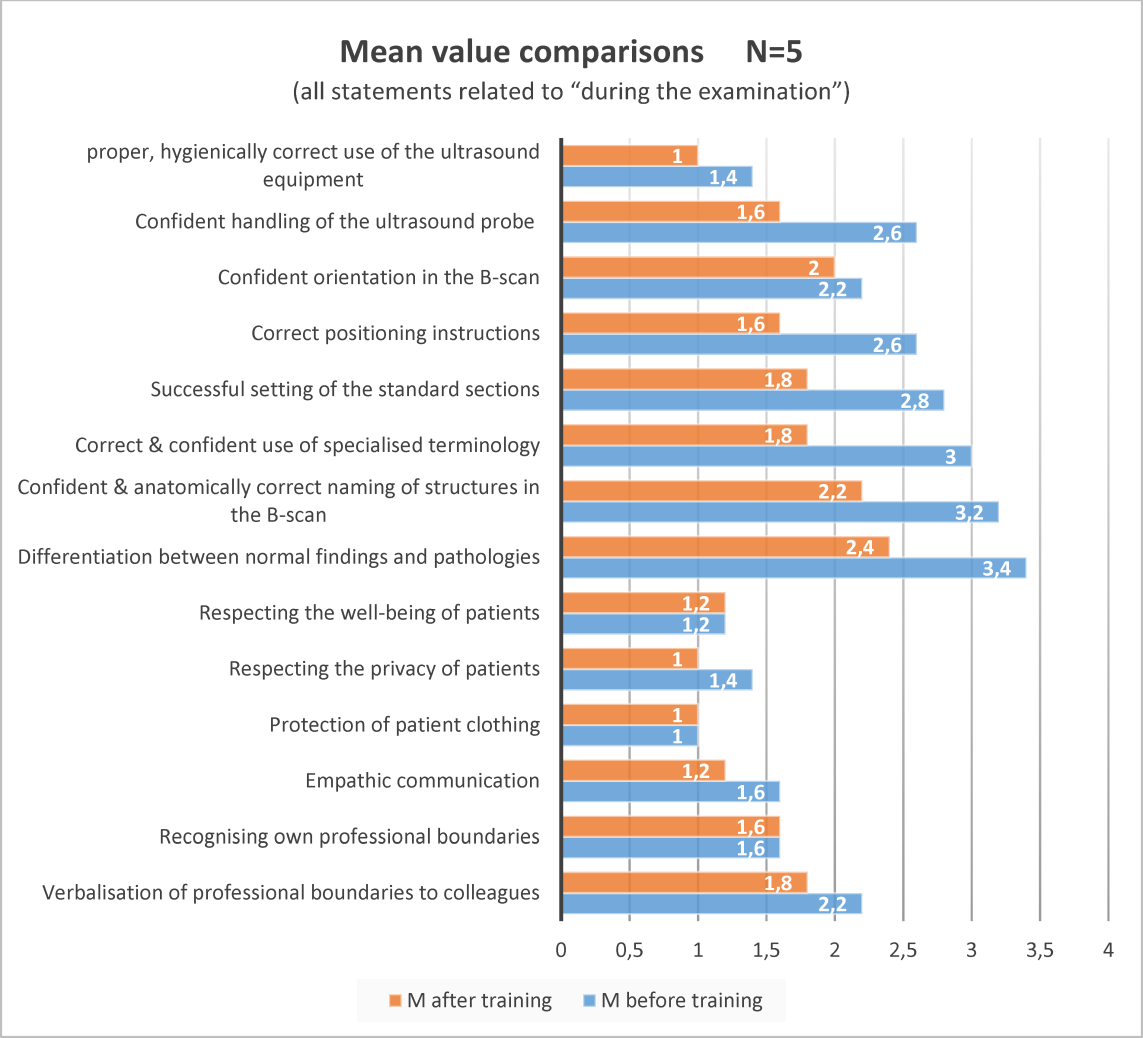
Mean change in agreement values for items relating to competence before/after SoSim training 0=applies, 4=does not apply

As part of the pilot program, all new recruits at the GYN at this point of time trained in transvaginal and transabdominal ultrasound with SoSim at determined and individually agreed training times. Two specific learning modules were defined to be completed during the course (see figure 1 (Fig. 1)). All modules contain a structured learning program with text and video-based learning aids, automatic error correction and audiovisual feedback. The five learners were monitored by means of cloud-based evaluation of the learning outcomes and accompanying self-assessment at a measurement point via online-based evaluation using the EvaSys software [https://www.electricpaper.biz//]. The survey included questions on demographics, previous experience, attitude, self-assessment of competences before and after training on the simulator, questions on general use of the simulator and transfer of learning to the clinic. The 14 competency questions were divided into technical (compliance with hygiene, probe handling, orientation in the B-scan, technical terms, setting standard sections, differentiation between normal and pathological findings), social (positioning, communication) and personal aspects (own limits, stress experience). The questions were answered using a five-point Likert scale from “applies” to “does not apply” (see figure 2 (Fig. 2)).

The piloting processes were analyzed and reflected in a circular manner by the authors by means of an exploratory survey of the perspectives of the participating authors and employees at MITZ in individual interviews. For this purpose, observation protocols and text documents were evaluated in the categories organizational-spatial, didactic-content, structural-personal.

## 3. Results

The process analysis confirmed research findings on self-directed learning to the effect that learners require learning assistance to reflect on their own learning process despite the availability of a comprehensive and transparent learning program with integrated learning management and feedback [2]. As part of the pilot, this was realized by preselecting and controlling the sequence of the learning modules to be completed with additional feedback from the supervising senior physician to enable prompt clinical applicability.

Furthermore, the greatest administrative effort for all those involved was required to coordinate individual appointments.

The response rate for the evaluation of SoSim utilization was 100%. The results for the competency questions in the evaluation of the five GYN career starters are shown in figure 2 (Fig. 2). The agreement values for the items increased for 11 of 14 questions in comparison before/after the training, while the values remained identical for three questions. 

## 4. Discussion

The results influence the further organization of structural framework conditions, the transfer of the project to other areas as well as the didactic support and education research to be established.

### Structural

For an acceptable use by learners, the SoSim-training as part of the overall training concept needs to be integrated into medical working hours, existing curricula, and further education programs [3]. Departments need to provide appropriate resources. In addition to standards for instruction and training specifications, the transfer of the learnt to the patient must be ensured.

To reduce the organizational effort, individual appointment booking for users was established using automated processes and digital booking tools.

### Transfer

Due to the positive changes in the approval ratings in the evaluation of SoSim use, the simulator will soon be accessible to medical staff in further training in the specialist areas of internal medicine and anesthesia. The learning modules to be completed will be determined according to learning requirements and clinical applicability. The SoSim will be successively integrated into student training and accompanied by educational research.

### Didactic support

Despite the integrated SoSim learning program, a learning assistant is required to pre-select and manage the learning modules. The results of the one-off evaluation indicate a subjective increase in learning. A larger sample with a pre-post design is aimed for significance tests and clear causal relationships.

## 5. Outlook

A transfer to further areas of education and the gradual implementation of SoSim training for student tutors and students is in planning and will be monitored via a separate study.

## Authors’ ORCIDs

- Anne Röhle: [0009-0001-6691-5401]
- Marie-Christin Willemer: [0009-0000-7950-5922]

## Competing interests

The authors declare that they have no competing interests.
